# Longitudinal Variation in Riparian Spider Communities Along Near‐Natural Streams

**DOI:** 10.1002/ece3.74512

**Published:** 2026-10-16

**Authors:** Léa Fargette, Martin H. Entling, Felicia Hoffmann, Alessandro Manfrin, Franziska Middendorf, Jens Schirmel

**Affiliations:** ^1^ iES Landau, Institute for Environmental Sciences RPTU University Kaiserslautern‐Landau Landau Germany; ^2^ Eusserthal Ecosystem Research Station RPTU University Kaiserslautern‐Landau Eusserthal Germany

**Keywords:** aquatic‐terrestrial interface, riparian habitat, river continuum concept, stream zonation, traits

## Abstract

Environmental conditions vary longitudinally along a river continuum, shaping the structure and functioning of biological communities, as explained by the River Continuum Concept. Riparian zones are important transitional areas linking the aquatic and terrestrial ecosystems. Therefore, a meta‐ecosystem perspective predicts that the longitudinal patterns described by the RCC extend into riparian habitats, leading to systematic change in terrestrial communities. We conducted a field study on near‐natural forested sections of five streams in southwestern Germany, sampling spiders in the up‐, mid‐ and downstream sections. We examined how variation in aquatic and terrestrial abiotic parameters among stream sections influences riparian spider diversity and community composition using pitfall traps and hand‐catches. Spider abundance increased along the stream course, whereas alpha diversity remained unchanged, suggesting that larger and more extensive riparian habitats in midstream and downstream reaches support higher population densities through greater habitat quality and resource availability without increasing local species richness. Despite similar levels of alpha diversity, each stream section harbored a distinct spider community composed of varying proportions of riparian specialists and habitat generalists. Correspondingly, spider trait composition changed markedly along the stream course: upstream reaches were characterized by heterogeneous communities with a high proportion of shade‐tolerant and web‐building species, whereas midstream reaches were more homogeneous and dominated by large‐bodied active hunters. Downstream communities were again more heterogeneous and contained many web‐building and open land‐preferring species. In contrast, spider moisture preferences remained largely unchanged along the stream course. Our findings enhance the understanding of how environmental longitudinal gradients shape riparian habitats and communities under near‐natural conditions, a fundamental step in understanding human impacts on riparian biodiversity.

Applying the River Continuum Concept to riparian zones, this study examined how longitudinal gradients in five southwestern German streams affect spider communities. While alpha diversity remained stable, spider abundance increased downstream, and community and trait compositions shifted significantly along the watercourse. These findings highlight how natural longitudinal variations structure riparian ecosystems, offering a crucial baseline for assessing human impacts on biodiversity.

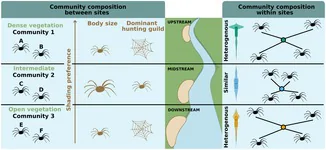

## 1 Introduction

Environmental conditions change progressively from a river's headwaters to its mouth, shaping the structure and functioning of biological communities. This longitudinal pattern is explained by the River Continuum Concept (RCC), which proposes a continuous relationship between shifts in physical and chemical parameters (e.g., nutrient inputs, width, depth, velocity, sediment load) and corresponding changes in biotic composition and ecosystem processes along the river gradient (Lalremsanga 2021; Vannote et al. 1980). Riparian zones along streams and rivers are ecologically important transitional ecosystems at the interface between terrestrial and aquatic environments. They are strongly shaped by hydrological influences, such as flow regimes, flooding and erosion (Henshall et al. 2011; Naiman et al. 2005). In addition, river secondary productivity affects riparian communities through variation in emergence fluxes, which modulates the availability of aquatic subsidies for riparian consumers (Kato et al. 2003; Middendorf, Bundschuh, et al. 2025; Wesner 2012). Together, these hydrologic dynamics and aquatic subsidies promote high levels of riparian biodiversity, supporting a mosaic of diverse communities that are adapted to recurrent disturbance regimes (Naiman et al. 1993). Given the strong influence of adjacent running water systems on riparian zones (Camporeale et al. 2013; Gething, Sarremejane, et al. 2026; Henschel et al. 2001), a meta‐ecosystem perspective is necessary to predict how stream longitudinal patterns extend into riparian habitats.

Riparian zones are heterogeneous habitats shaped by local environmental conditions and adjacent rivers, resulting in variation both laterally and longitudinally (Bates et al. 2013; Doretto et al. 2020; Middendorf, Eitzinger, et al. 2025). Laterally, gradients in temperature, humidity, inundation frequency, and availability of aquatic food resources can influence species distributions and microhabitat preferences in arthropod groups such as carabid beetles (Bates et al. 2013; Paetzold et al. 2005). In addition, the lateral extent and characteristics of riparian habitats can vary seasonally with cycles of flooding and drying, which may expose dry riverbeds supporting terrestrial arthropod communities distinct from those of adjacent riparian zones (Sánchez‐Montoya et al. 2016; Sánchez‐Nogueras et al. 2025; Steward et al. 2011).

Beyond such lateral patterns, the longitudinal position along a stream course may itself shape riparian communities through systematic changes in environmental conditions. Longitudinally, bank morphology and flood regimes change, influencing the connectivity between water and land (Ramey and Richardson 2017; Tomanova et al. 2007). Upstream reaches are typically characterized by narrow riparian areas, steep banks, and higher erosion, resulting in lower connectivity, with floods mainly driven by direct rainfall and snowmelt. In contrast, midstream and downstream reaches tend to have wider riparian areas and lower banks, leading to higher connectivity, with floods that more strongly shape riparian habitats. In addition, the emergence of aquatic insects is expected to increase along the river course (Ramey and Richardson 2017; Vannote et al. 1980), potentially influencing riparian predator communities, which may shift from predominantly terrestrial‐feeding species to host more aquatic‐feeding species (Kato et al. 2004; Twining et al. 2021). These longitudinal gradients are expected to have ecological consequences affecting riparian arthropod communities. Eyre, Lott, and Luff (2001); Eyre, Luff, and Phillips (2001) compared carabid beetle and staphylinid beetle assemblages across 179 sites spanning two highland river catchments and two lowland river catchments in Scotland and Northern England. They found changes in carabid assemblages along gradients of sediment types, vegetation cover, humidity, and shading but not specifically along the upstream–downstream gradient. In contrast, changes in staphylinid assemblages were associated with the same environmental gradients and also reflected the longitudinal gradient. Framenau et al. (2002) compared riparian arthropod diversity between upper and lower floodplains of ten rivers in south‐east Australia, along an altitudinal gradient closely associated with longitudinal river position. Their results indicated taxon‐specific responses, with spider densities decreasing downstream, while carabid beetles were generally less abundant and exhibited weaker longitudinal patterns. However, a similar study on spiders in the European Alps found no change in spider abundance along the stream course (Manderbach and Framenau 2001). Finally, longitudinal variation in stream conditions may influence lateral connectivity and the distribution of riparian taxa. For example, Gething, Sarremejane, et al. (2026) found that different water levels associated with the longitudinal stream position affected the abundance of water‐associated species but not the cross‐channel community similarity between opposing banks. Overall, these results suggest that riparian arthropod responses to longitudinal environmental gradients are taxon‐ and region‐specific, although conclusions remain limited by the scarcity of comparable studies. Instead, most existing studies on riparian arthropods have focused on the comparison between sites located upstream and downstream of discrete disturbances rather than examining gradual changes along the river continuum. For example, Jackson et al. (2016) found that the presence of an invasive fish species resulted in dietary overlap with riparian spiders, which was associated with a reduced spider abundance at downstream sites where fish were present compared to upstream fishless sites.

Among riparian arthropods, spiders (Araneae) are a key group to understand the influence of longitudinal gradients extending from the river channel into riparian zones. As dominant predators, they play a functionally important role in food webs (Churchill 1997) and exhibit substantial functional diversity in foraging strategies, prey range, and vertical habitat use (Cardoso et al. 2011). Moreover, many riparian spiders (e.g., Tetragnathidae) rely on emerging aquatic insects, so that variation in river secondary productivity and aquatic insect fluxes may indirectly shape spider communities through diet shifts (Middendorf, Bundschuh, et al. 2025). Spider diversity and community composition are highly sensitive to environmental variation, particularly to changes in microclimatic and microhabitat conditions (Gallé et al. 2024; Ziesche and Roth 2008) as well as prey availability (Spears and MacMahon 2012). Accordingly, changes in species composition along environmental gradients are also likely to be accompanied by shifts in community trait composition. Communities are expected to shift from shade‐ and dry‐tolerant species upstream to more open‐ and moisture‐preferring species in downstream reaches (Eyre et al. 2002; Gething, Hayes, et al. 2026), as canopy openness and flooding frequency increase along the stream continuum, following the RCC (Doretto et al. 2020; Vannote et al. 1980). Mobility and hunting strategies are also expected to vary along the stream continuum; more mobile free‐hunting species may be associated with the more flood‐prone and open downstream reaches (Lafage et al. 2015; Pedley and Dolman 2014; Schirmel et al. 2016).

Understanding general longitudinal gradients in riparian communities is essential for assessing the effects of human impacts on riparian biodiversity. We therefore studied riparian spider communities at near‐natural, forested sections of five streams in Rhineland‐Palatinate, southwestern Germany. Spiders were sampled in the up‐, mid‐ and downstream sections of each stream. We further measured a suite of aquatic and terrestrial parameters to characterize differences among stream sections and to relate spider community patterns to local habitat conditions. We hypothesized that (i) different stream sections host distinct species compositions due to taxa responding differently to changing longitudinal stream conditions. We further hypothesized that (ii) abundance and diversity increase from upstream to downstream, and that (iii) communities shift downstream towards more active hunting species relative to web builders, larger‐bodied species, higher moisture preference, and lower shading preference. Finally, we expected (iv) within‐site spatial variation (multivariate dispersion) of communities to increase along the longitudinal gradient in association with increasing habitat heterogeneity.

## 2 Materials and Methods

### 2.1 Study Area

The sampling was conducted in May and September 2023 in Rhineland‐Palatinate, southwestern Germany. We studied five streams in the Upper Rhine Valley: the third‐order Speyerbach, Queich, Otterbach, and Lauter/Wieslauter, and the second‐order Heilbach (Strahler stream order), all originating in or near the Palatinate Forest and flowing into the Rhine. For each stream, three near‐natural riparian sampling sites were selected in the up‐, mid‐, and downstream sections. All sampling sites were located in protected areas, either within the UNESCO Biosphere Reserve Palatinate Forest–North Vosges or within Natura 2000 sites (Figure 1). To minimize the influence of agricultural and urban land use, all sites were located within forests. Sites were selected based on near‐natural shoreline characteristics, including gentle slopes and the absence of anthropogenic bank reinforcement. For full field site details and site selection methods, see Middendorf, Eitzinger, et al. (2025).

**FIGURE 1:**
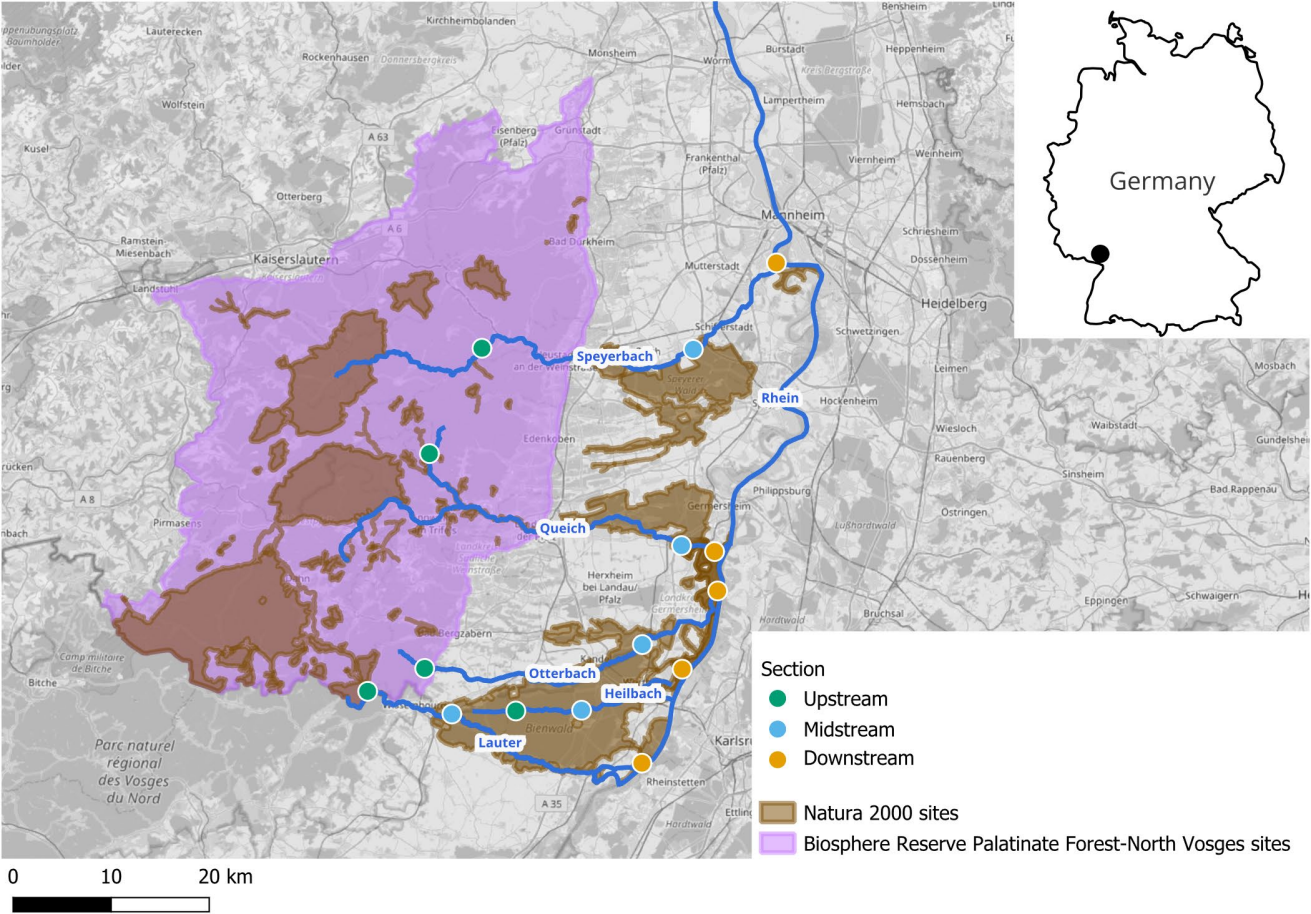
Map of the study area and the 15 sampling sites including the stream section they correspond to and the presence of protected areas.

### 2.2 Site Environmental Characterization

At each site, we measured ten terrestrial environmental parameters at the riparian bank, namely the length and width of the riparian area (m), bank slope, vegetation height at the sampling site (cm), proportions of bare ground, herb, canopy cover, soil pH, soil moisture, and the proportion of coarse soil fraction (2–4 mm) (see also Middendorf, Eitzinger, et al. 2025). Additionally, four parameters were measured in the stream using a WTW field portable probe, namely water pH, temperature, and dissolved water oxygen (in % saturation and concentration, mg/L).

### 2.3 Spider Sampling and Processing

Spiders were sampled two times in 2023, from 8 to 19 May and from 11 to 21 September, to capture temporal variation in spider assemblages and seasonal differences in community composition (Table S1). At each site and for each sampling event, spiders were collected using ten pitfall traps deployed along the riparian area in two rows of 5 traps, one 0.5 m away from the water edge and one 1.5 m away. The distance from the water's edge was determined based on the water level in May, and traps were placed in the same areas in September, despite the water level having decreased at several sites. The traps were deployed for 7 days, in both May and September.

Additionally, standardized hand‐catches were conducted at each site for 2 h in May and 1.5 h in September to target vegetation‐dwelling species that are underrepresented in pitfall traps (Middendorf, Eitzinger, et al. 2025). The decision to decrease the sampling time by half an hour between sampling points was made due to increased experience and efficiency of the samplers.

Adult spider specimens were identified to species level using the identification book of M. J. Roberts (1995), and the digital identification key of Nentwig et al. (2025). Spiders from the Linyphiidae family were determined to species level using M. J. Roberts (1993) and identifications of specimens from the cryptic genus *Porrhomma* were verified by an expert. Female spiders of the genus *Trochosa* were sorted to species using the epigyne photographs from Hepner and Milasowszky (2006) and a subset of specimens was dissected to examine the genitals and confirm the identifications. As all confirmed specimens were correctly identified, the remaining individuals were considered reliably identified based on epigyne morphology alone.

Trait information was collected for all species across four trait categories: shading preference as a proxy for temperature preference (numerical value between 0 and 1, ranging from preference for open to shaded habitats) and moisture preference (numerical value between 0 and 1, ranging from moist to dry habitats), both obtained from Entling et al. (2007); body size (female body length in cm) from Nentwig et al. (2025); and hunting guild (categorical: active hunter or web builder) from Gloor et al. (2017) (Table S2).

### 2.4 Diversity Estimates and Functional Traits

Changes in abundance were assessed as differences in the total number of hand‐caught and pitfall‐trapped individuals between sites. For pitfall traps, this measure represents activity density but is referred to as abundance for consistency.

Taxonomic alpha diversity was estimated by calculating Hill numbers (Geres et al. 2025). Using the *estimateD* function from the *iNEXT* package (version 3.0.2, Hsieh et al. 2016) for R (version 4.4.2), we estimated three species diversity metrics (Hill numbers), by increasing the importance of species' abundance (increase in parameter *q*). For *q* = 0, the estimate reflects species richness (equal weight for all species), for *q* = 1, the estimate reflects the Shannon diversity index (species weighted by relative abundance), and for *q* = 2, the estimate is the inverse of the Simpson diversity index (species with high abundance are given greater weight). These diversity variables will be further referred to as species richness (for *q* = 0), Shannon diversity (for *q* = 1), and Simpson diversity (for *q* = 2).

Changes in continuous functional traits (i.e., shading preference, moisture preference and body length) were estimated by computing the community weighted mean (CWM) for each sampling site. This corresponds to the average trait value in the community, weighted by the relative abundance of each species. For the categorical trait hunting guild, we measured the proportion of each category (ground hunter or web builder) within the community.

### 2.5 Statistical Analysis

In a first step, we performed two Principal Component Analyses (PCA) using the package *FactoMineR* (version 2.11, Lê et al. 2008) in R (version 4.5.2, R Core Team 2025) to examine the relationships between stream sections and terrestrial and aquatic environmental variables, respectively. The analyses were conducted separately to account for the correlations between aquatic and terrestrial parameters (Table S3) while evaluating their independent variation across stream sections.

Subsequent analyses of spiders were performed separately for pitfall and hand‐catch samples (hereafter referred to as the pitfall dataset and the hand‐catch dataset). To assess the effect of stream section on site‐level spider communities (hypotheses i–iii), May and September samples were pooled within each site for both datasets. The ten pitfall traps within each site were treated as non‐independent spatial replicates and pooled. This resulted in one pooled pitfall and one pooled hand‐catch sample per site (*n* = 15 each).

Redundancy analyses (RDA) from the package *vegan* (version 2.6‐10, Oksanen et al. 2025) for R were conducted to determine the effect of stream section on site‐level spider species composition (hypothesis i). Analyses were conducted on the Hellinger‐transformed community matrix, with stream identity included as a conditioning variable (partial RDA). For each RDA, the significance of stream section and RDA axes was assessed using a permutation test for constrained correspondence analysis using the *anova.cca* function. Indicator species analyses were conducted using the *indval* function of the *labdsv* package (version 2.3.1; D. W. Roberts 2026) to identify species significantly associated with individual stream sections.

To assess differences among stream sections in site‐level abundance and diversity (as species richness, Shannon diversity and Simpson diversity using Hill numbers; hypothesis ii), as well as functional traits (as CWM of shading preference, moisture preference, body length and % of hunting guild categories; hypothesis iii), we used generalized linear mixed‐effects models (GLMMs) from the package *glmmTMB* (version 1.1.13, McGillycuddy et al. 2025), and linear mixed‐effects models (LMMs) from the package *lme4* (version 1.1.37, Bates et al. 2015). All the models included stream section as a fixed factor and stream identity as a random factor. For diversity variables and continuous functional traits, LMMs were fitted with a Gaussian distribution on raw data or log‐transformed data (depending on AIC and model residuals diagnostics). For hunting guild, a betabinomial GLMM was fitted to account for overdispersion and mild zero‐inflation, with model selection guided by residual diagnostics. For abundance, a Generalized Poisson GLMM was fitted to account for overdispersion (Noriszura and Aziz Jemain 2007), with model selection guided by AIC and residual diagnostics. For the pitfall dataset, trap loss resulted in unequal sampling effort between sites (6–10 successful traps per sites in May, 9–10 in September, and 16–20 overall after pooling sampling periods). We retained all functional traps and included an offset term in the (G)LMMs to account for unequal sampling effort. For each model, significance of stream section was assessed using a Wald Chi‐square test (Type‐II ANOVA) from the package *car* (version 3.1‐3, Fox et al. 2023). Where stream section had an overall effect, pairwise post hoc comparisons of estimated marginal means were conducted using the package *emmeans* (version 2.0.1, Lenth et al. 2026), with *p*‐values adjusted using the Tukey method for multiple comparisons.

In contrast to the site‐level analyses, within‐site spatial variation in spider community composition (hypothesis iv) was assessed for the pitfall trap dataset at the individual pitfall‐trap level using a distance‐based test of homogeneity of multivariate dispersions (Anderson 2006). Because traps with zero captures provide no information on community variation, only traps with non‐zero captures were retained. Consequently, September samples were excluded because traps were not placed at identical micro‐locations between sampling events (precluding pooling), and no individuals were captured at two sites; the number of traps with captured individuals per site ranged from *n* = 3–10 in May and *n* = 0–5 in September (Figure S1). Although multivariate dispersion was only weakly correlated with the number of pitfall traps (*r* = −0.27, *p* = 0.164), we used a bootstrap approach with 500 iterations, randomly selecting three traps per site in each iteration to account for unequal numbers of traps among sites. Bray–Curtis dissimilarities were calculated among traps, and the mean distance of traps to their site centroid in multivariate space was computed using the *betadisper* function from the *vegan* package. Site‐level mean distances were then compared among stream sections computing a permutation‐based ANOVA using the *aovp* function from the *lmPerm* package (version 2.1.6, Wheeler and Torchiano 2025).

For all analyses, results with *p* < 0.1 were considered as potential trends, while results with *p* < 0.05 were considered statistically significant.

## 3 Results

### 3.1 Variation in Environmental Parameters Across Stream Sections

The PCA of terrestrial environmental variables (Figure 2a) revealed clear gradients along the first two axes, which explained 40.0% (Dim1) and 24.4% (Dim2) of the total variation. Dim1 was primarily associated with vegetation structure and soil characteristics. Vegetation height, herb layer cover, and soil moisture were negatively correlated with Dim1, whereas canopy cover, open ground cover, and bank slope were positively associated. Dim2 reflected variation positively related to riparian area width and length, as well as the proportion of coarse soil fraction and pH, whereas bank slope was negatively associated. Sites from up‐, mid‐, and downstream sections formed partially separated clusters along Dim2, indicating spatial differentiation in riparian dimensions and soil texture along the stream gradient (see Table S4 for correlation significance).

**FIGURE 2:**
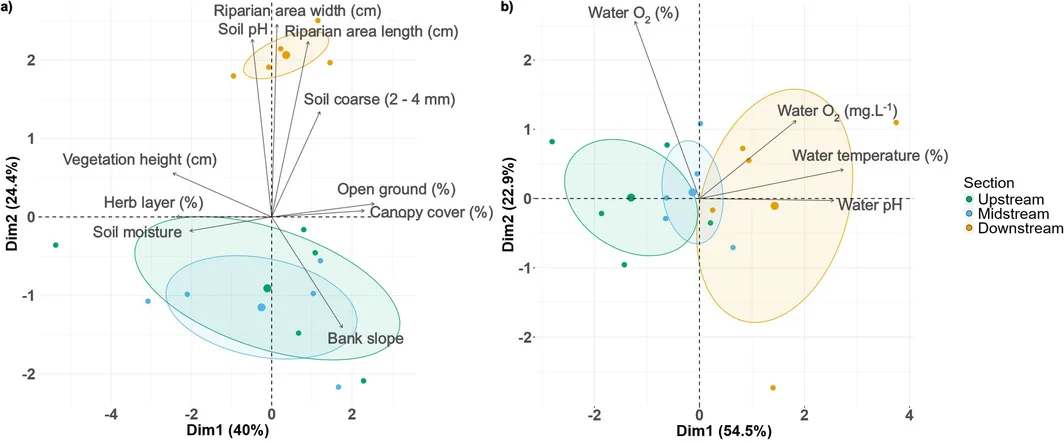
PCA biplots of (a) terrestrial and (b) aquatic environmental variables. Points represent sampling sites and are grouped by stream section.

In the PCA of aquatic environmental variables (Figure 2b), the first two axes respectively explained 54.5% (Dim1) and 22.9% (Dim2) of the total variation. Water temperature, water pH, and quantity of dissolved oxygen were positively associated with Dim1. Dim2 reflected a positive relation with the proportion of dissolved oxygen. Sites from up‐, mid‐, and downstream sections formed partially separated clusters along Dim1, indicating spatial differentiation in water characteristics along the stream gradient (see Table S4 for correlation significance).

### 3.2 General Spider Results

In total 1509 individuals belonging to 107 species across 23 families were collected in the 15 sites. 778 individuals across 67 species were sampled with pitfall traps and 731 individuals across 84 species were collected by hand (Tables S5 and S6). The most diverse family was Linyphiidae with 45 distinct species, followed by Lycosidae with 11 distinct species. Overall, *Piratula hygrophila* (*n* = 350), *Pardosa saltans* (*n* = 216), *Tenuiphantes flavipes* (*n* = 121) and *Pardosa amentata* (*n* = 99) were the most abundant species (Table S7). More specifically, *P. hygrophila* (24.81%), *P. saltans* (20.44%) and *P. amentata* (10.15%) dominated in the pitfall samples and *P. hygrophila* (21.48%), *T. flavipes* (10.67%), and *P. saltans* (7.80%) dominated in the hand‐caught samples (Table S7). Down‐ and midstream spider communities were dominated by *P. hygrophila* (downstream: 26.35%, midstream: 30.32%) whereas the upstream community was dominated by *P. saltans* (33.65%) (Table S7).

### 3.3 Effects of Stream Section on Site‐Level Community Composition

Species composition significantly varied across stream sections both for the pitfall (partial RDA, adj. *R* ^2^ = 0.135, *F* = 1.78, *p* = 0.028) and hand‐catch datasets (partial RDA, adj. *R* ^2^ = 0.136, *F* = 1.82, *p* = 0.013). The first canonical axis was significant (pitfall dataset: *p* = 0.029; hand‐catch dataset: *p* = 0.044), whereas the second axis was not (pitfall dataset: *p* = 0.444; hand‐catch dataset: *p* = 0.136) and explained comparatively little variation. The first axis can therefore be interpreted as the section gradient from upstream to downstream for both datasets.

Different species tended to cluster with different stream sections. Notably, most Lycosidae species clustered with mid‐ or downstream sections. *Piratula hygrophila* tended to occur between the downstream and midstream sections, whereas *Pardosa saltans* clustered mainly with the midstream section. In contrast, *Pardosa lugubris* and *P. hortensis* tended to cluster with the downstream section. *P. amentata* mainly clustered with the downstream section in the pitfall dataset, but with both the midstream and downstream sections in the hand‐catch dataset. In the genus *Trochosa*, *T. terricola* clearly clustered with the upstream section for both datasets, whereas *T. ruricola* clustered more with mid‐ and downstream sections (Figure 3a,b).

**FIGURE 3:**
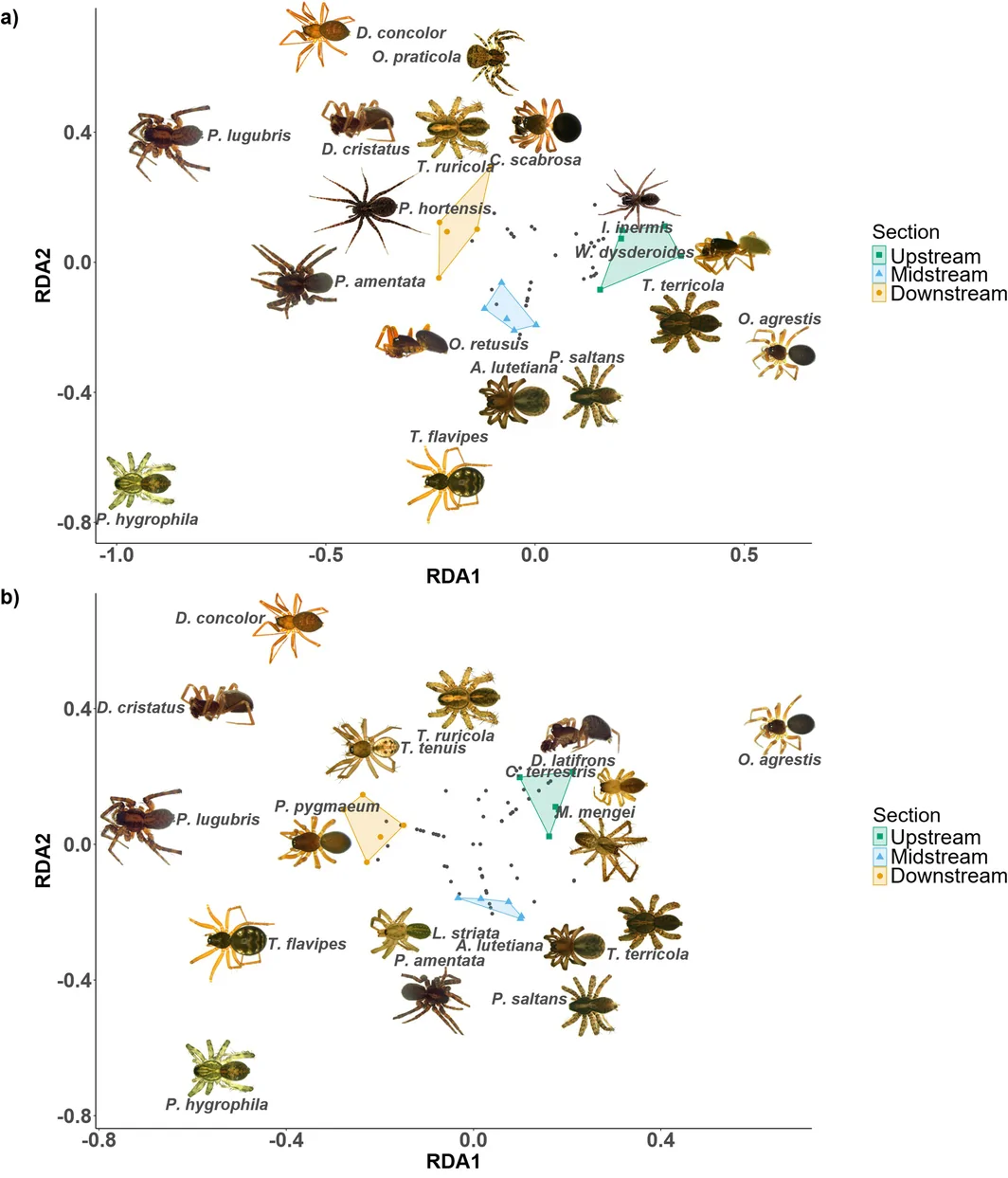
Community composition of spiders in the three longitudinal sections of five streams based on (a) the pitfall traps and (b) the hand‐catches. RDA of Hellinger‐transformed individual numbers per spider species in each of the 15 sampling sites. For readability, only the top contributing species are labeled for clarity (those exceeding the 75th percentile of Euclidean distance from the ordination origin in the pitfall dataset and the 80th percentile in the hand‐catch dataset); all others are shown as gray points (Spiders pictures: Oger 2026).

Within the family Linyphiidae, species belonging to the same genus also tended to cluster with different sections. *D. latifrons* and *T. tenuis* clustered with the midstream section (hand‐catch dataset) whereas *Diplocephalus cristatus* and *Tenuiphantes flavipes* clustered with the downstream section. *Oedothorax agrestis* clearly clustered with the upstream section, whereas *O. retusus* clustered more with the midstream section (pitfall dataset) (Figure 3a,b).

Four species for each dataset were identified as indicators of a section. For the pitfall dataset, *Diplostyla concolor* (IndVal = 0.89, *p* = 0.008), *Diplocephalus cristatus* (IndVal = 0.64, *p* = 0.067), and *Ozyptila praticola* (IndVal = 0.62, *p* = 0.044) were associated with the downstream section, while *Piratula hygrophila* (IndVal = 0.65, *p* = 0.068) was associated with the midstream section. In the hand‐catch dataset, *Diplostyla concolor* (IndVal = 0.81, *p* = 0.009), *Pardosa lugubris* (IndVal = 0.68, *p* = 0.071), *Porrhomma pygmaeum* (IndVal = 0.60, *p* = 0.072), and *Diplocephalus cristatus* (IndVal = 0.58, *p* = 0.067) were all associated with the downstream section.

### 3.4 Effects of Stream Section on Abundance, Diversity, and Functional Traits

Significant differences among stream sections were found in abundance for both the pitfall dataset (*χ* ^2^ = 9.383, *p* = 0.009) and the hand‐catch dataset (*χ* ^2^ = 21.029, *p* < 0.001). In both cases, the total number of individuals was higher in the midstream and downstream sections (pitfall dataset: estimated difference between down and upstream = 0.745, *p* = 0.098; mid vs. upstream = 1.077, *p* = 0.006; hand‐catch dataset: down vs. upstream = 0.602, *p* < 0.001; mid vs. upstream = 0.658, *p* < 0.001), with approximately twice as many individuals recorded compared to the upstream section (Figure 4a,b). No significant differences were detected for any of the diversity estimates in either the pitfall dataset (Hill numbers q = 0: *χ* ^2^ = 3.672, *p* = 0.160; *q* = 1: *χ* ^2^ = 0.592, *p* = 0.744; *q* = 2: *χ* ^2^ = 1.328, *p* = 0.515) or the hand‐catch dataset (*q* = 0: *χ* ^2^ = 2.862, *p* = 0.239; *q* = 1: *χ* ^2^ = 3.037, *p* = 0.219; *q* = 2: *χ* ^2^ = 3.325, *p* = 0.190).

**FIGURE 4:**
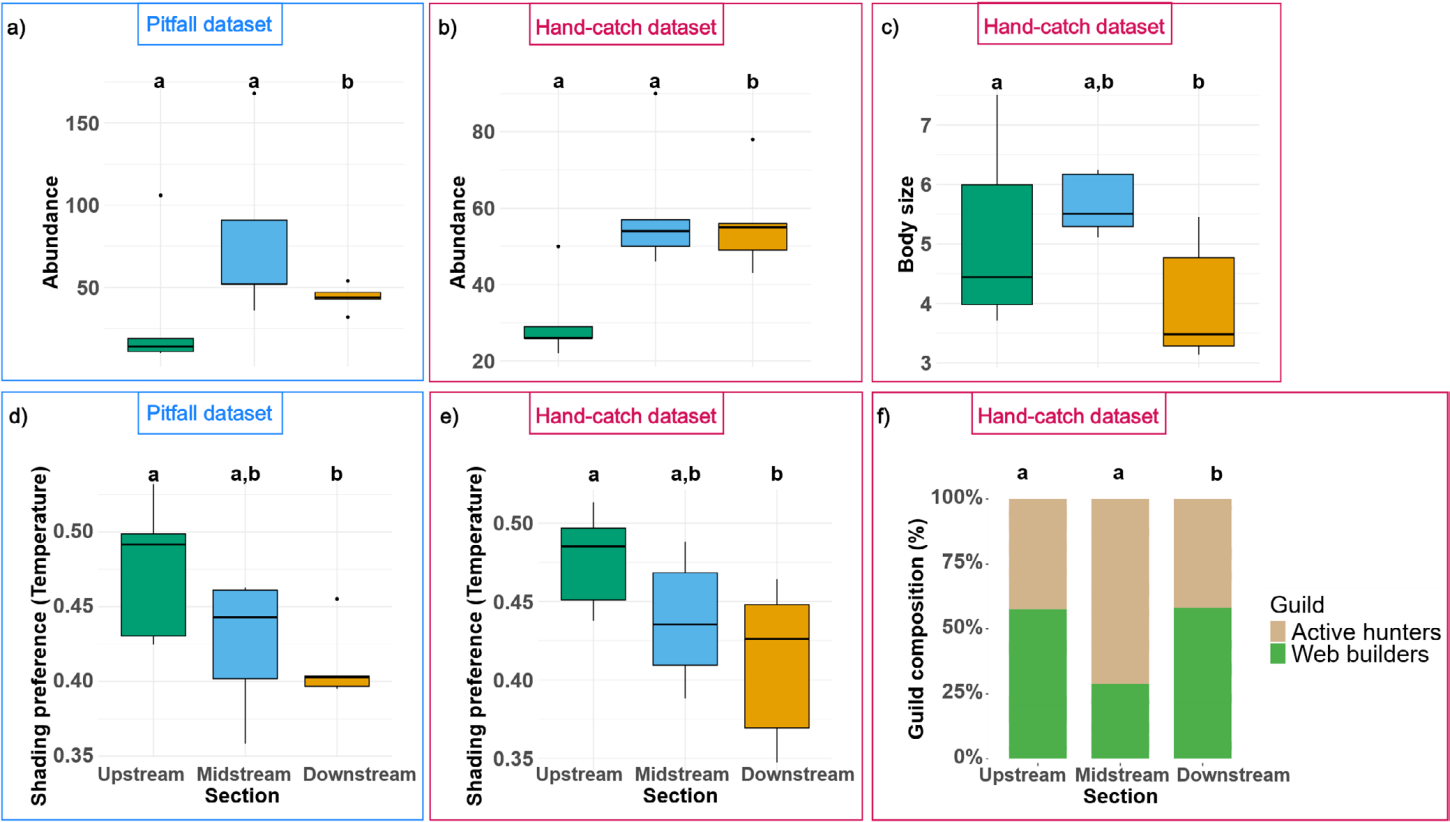
Differences among up‐, mid‐, and downstream sections in pitfall data: (a) total abundance, (d) shading preference (as CWM values); and hand‐catch data: (b) total abundance, (c) body size (as CWM values), (e) shading preference (as CWM values), (f) guild composition. Different lowercase letters represent pairwise differences or trends (*p* < 0.1) between river sections based on Tukey's post hoc tests, while shared letters indicate no difference.

The CWM of shading preference differed significantly among stream sections in both the pitfall dataset (*χ* ^2^ = 7.728, *p* = 0.021) and the hand‐catch dataset (*χ* ^2^ = 6.267, *p* = 0.044), decreasing from up to downstream (pitfall dataset: down vs. upstream = −0.064, *p* = 0.067; hand‐catch dataset: down vs. upstream = −0.066, *p* = 0.086) (Figure 4d,e). The CWM of body size significantly varied across stream sections in the hand‐catch dataset (*χ* ^2^ = 10.031, *p* = 0.007), decreasing from mid‐ to downstream (down vs. midstream = −1.638, *p* = 0.035) (Figure 4c), but not in the pitfall dataset (*χ* ^2^ = 0.110, *p* = 0.946). The proportion of active hunters differed significantly between sections in the hand‐catch dataset (*χ* ^2^ = 7.912, *p* = 0.019): the proportion of active hunters was lowest midstream and similar at up‐ and downstream sites (down vs. midstream = −1.206, *p* = 0.031; mid vs. upstream = 1.182, *p* = 0.045) (Figure 4f). Differences in hunting mode were not significant in the pitfall dataset (*χ* ^2^ = 3.231, *p* = 0.199). No significant differences were detected for the CWM of moisture preference in either the pitfall dataset (*χ* ^2^ = 1.929, *p* = 0.381) or the hand‐catch dataset (*χ* ^2^ = 0.887, *p* = 0.642).

### 3.5 Effects of Stream Section on Within‐Site Spatial Variation

Distances to site centroid differed significantly between sections (99% of all bootstraps with permutation ANOVA, *p* < 0.05). However, the magnitude of these differences was small (mean root mean square = 0.034), indicating only minor variation in how similar samples were within sites. Overall, samples from the midstream section were more similar to each other (i.e., more homogeneous in species composition), whereas samples from the upstream section were more variable (i.e., more heterogeneous), with the downstream section showing an intermediate pattern (Figure 5).

**FIGURE 5:**
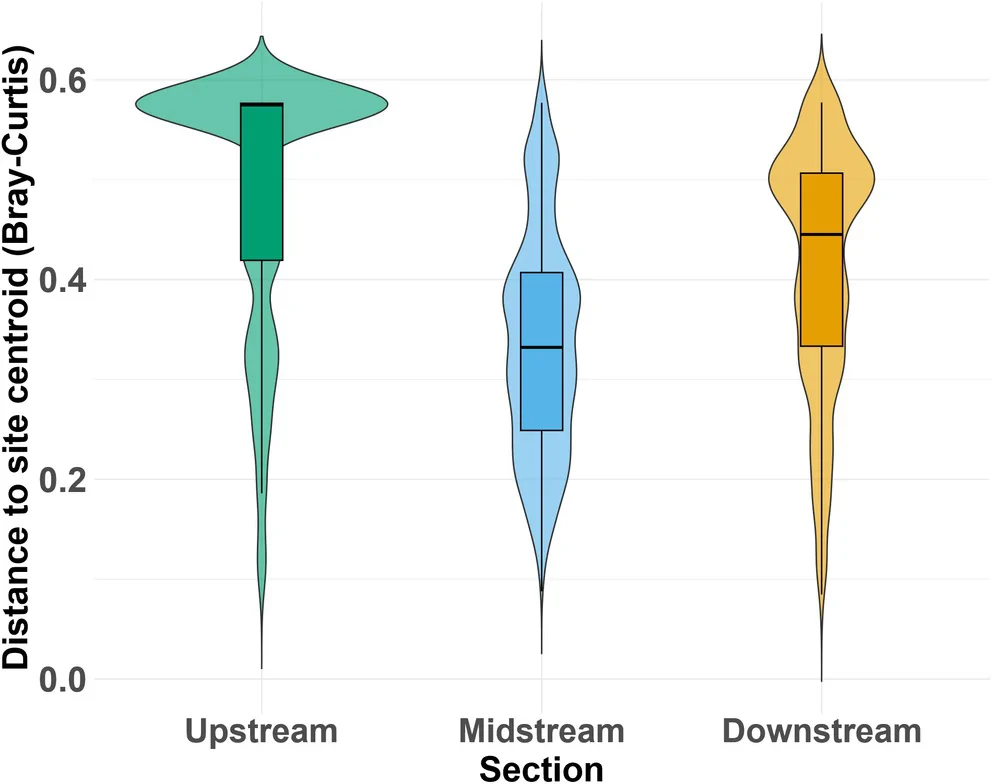
Comparison of average distances to the site centroid for communities sampled by the pitfall traps at each site, across stream sections.

## 4 Discussion

As hypothesized, spider abundance increased longitudinally from up‐ to mid‐ and downstream sections in both datasets. In contrast to our expectations, alpha diversity did not change significantly across stream sections. The higher abundance observed in midstream and downstream sections may reflect improved habitat quality and greater resource availability associated with the wider and more extensive riparian habitats typically found downstream, supporting larger populations without a corresponding increase in alpha diversity. Hydrological disturbance may provide an additional mechanism, as experimental flooding has been shown to increase spider abundance, species richness, and the occurrence of riparian specialists (Mutel et al. 2026). A similar longitudinal increase in abundance has been reported for carabid beetles, another important group of riparian predators (Höppner and Hering 1997; Middendorf, Eitzinger, et al. 2025). However, available studies on riparian spiders found no change or even a decrease in abundance along the stream course (Framenau et al. 2002; Manderbach and Framenau 2001). Interestingly, this contrast between carabids and spiders is also apparent in studies comparing different riparian habitat types, where floodplain habitats supported higher carabid richness and abundance but not higher spider abundance or richness (Bauspiess et al. 2024). Together, these contrasting findings may reflect differences in spider community composition among regions. Responses to emerging aquatic insects are generally strongest and most consistent for web‐building spiders, whose abundance is often positively associated with emergence fluxes (Kato et al. 2003; Middendorf, Bundschuh, et al. 2025; Wesner 2012). In contrast, responses of free‐hunting and ground‐dwelling spiders are less consistent, suggesting a more context‐dependent reliance on aquatic subsidies (Middendorf, Bundschuh, et al. 2025). This context dependency may also reflect differences in the pathways through which aquatic subsidies reach terrestrial consumers, as crawling or shore‐emerging aquatic insects can provide an important resource for ground‐dwelling spiders, whereas surface‐emerging prey may be more readily exploited by web‐building spiders (Kowarik et al. 2023). Variation among studies may therefore reflect differences in the relative dominance of these functional groups across riparian spider communities. In contrast, despite the apparent increase in habitat quality and resource availability downstream, the lack of longitudinal variation in alpha diversity suggests that the number of available ecological niches may be comparable among stream sections. This pattern was consistent across all Hill numbers (q = 0, 1, 2). It differs from the increase in species richness along the stream continuum reported for carabid beetles in the same region (Middendorf, Eitzinger, et al. 2025).

As expected, spider species composition differed among stream sections, indicating pronounced turnover along the longitudinal gradient consistent with changes in environmental conditions, particularly canopy cover. This was reflected in shifts within the most abundant genera, from more shade‐associated species upstream or midstream to more open‐tolerant species towards downstream, for example, *T. terricola* upstream to *T. ruricola* downstream, *D. latifrons* midstream to *D. cristatus* downstream, and *O. agrestis* upstream to *O. retusus* downstream, as reported by Entling et al. (2007). Comparable shifts in species composition have been reported for wolf spiders along altitudinal stream gradients in Australia (Framenau et al. 2002) and for carabid beetles along the same streams as in the present study (Middendorf, Eitzinger, et al. 2025). In line with these compositional changes, functional trait composition also varied among stream sections. Overall, this variation in functional traits reflects underlying environmental gradients shaping community assembly through environmental filtering and local habitat conditions, further influenced by broader landscape characteristics. In particular, the spiders' shading preference decreased downstream, consistent with the RCC predictions of increasingly open riparian conditions along the stream course (Doretto et al. 2020; Vannote et al. 1980). This pattern aligns with regional differences observed in this study, ranging from forested upstream areas to the more open Rhine Valley downstream. However, these longitudinal differences were not fully reflected by the local environmental variables measured directly at each site. We did not detect clear evidence of higher canopy cover upstream and even observed a slight increase in vegetation height downstream, which may provide refuges for spiders and reduce the effect of solar radiation (Laino et al. 2025). Thus, the observed shift in shading preference likely reflects broader longitudinal differences in riparian landscape structure that were not adequately captured by our site‐level measurements, highlighting the importance of considering multiple spatial scales when interpreting patterns in community and trait composition. In contrast, moisture preference did not vary along stream sections, consistent with the absence of changes in measured soil moisture. This contrasts with evidence that changes in water level availability can shift communities from water‐associated taxa to more generalist species (Gething, Sarremejane, et al. 2026), suggesting that the relatively stable soil moisture conditions across our sites may have limited such changes in moisture preference. Together, these results suggest that longitudinal variation in riparian shading was strong enough to influence spider shading preference, whereas hydrological differences along the stream course were insufficient to drive shifts in moisture‐related traits.

We further detected differences in body size and hunting strategy among stream sections, although these patterns were only evident from the hand‐catch dataset. Interestingly, body size peaked in midstream sections, contrary to our expectation that downstream reaches—with larger habitats and coarser substrate—would support larger prey and higher prey densities, thereby favoring large‐bodied predators with greater space requirements (Framenau et al. 2002). However, downstream reaches are also wider, flatter, and more prone to flooding (Lambeets et al. 2008), which may favor smaller, more mobile species that are better adapted to frequent disturbance (Entling et al. 2011; Manderbach and Framenau 2001). In this context, the peak body size observed in midstream sections may reflect a trade‐off between increasing habitat and resource availability downstream and the disturbance associated with floods. Changes in feeding guilds also reflect environmental filtering, and we observed the highest proportion of web builders in upstream and downstream sections. Web‐building spiders are generally expected to benefit from less disturbed, forested conditions typical of upstream floodplains (Ramberg et al. 2020; Schirmel et al. 2016). Interestingly, downstream sections also supported a relatively high proportion of web builders compared to active hunters, likely reflecting the increased vegetation height and wider riparian zones, which provide more structural habitat and support higher densities of vegetation‐dependent web‐building spiders (Pires et al. 2022).

Finally, despite similar overall diversity patterns, we observed differences in the within‐site spatial variation, with communities being compositionally most similar in midstream section. This pattern coincided with the highest proportion of large‐bodied, active hunting spiders and may therefore reflect differences in functional community assembly along the longitudinal gradient. Longitudinal changes in community composition are not always monotonic and the relative importance of mechanisms structuring communities (e.g., environmental filtering, biotic interaction, stochasticity) can vary along the stream continuum (Bao et al. 2026). In our study, the higher compositional similarity midstream, may partly reflect more uniform habitat conditions, compared to the structurally complex and more vegetated upstream and downstream sections, which may promote greater variation in species composition and traits. However, the concomitant shift in functional trait composition suggests that the midstream pattern may not be explained by habitat complexity alone but could also reflect changes in the environmental filtering or biotic processes structuring these communities along the river.

## 5 Conclusion

In this study, we detected clear differences in both community structure and trait composition among near‐natural sections of five streams. A key result was a pronounced longitudinal shift in community composition, mirrored by corresponding changes in functional traits—most notably a transition in shading preference from shade‐associated species to those that prefer more open habitats. At the same time, variation in community similarity within stream sections suggests that local habitat heterogeneity also shaped community assembly, with more homogeneous communities occurring midstream, where large‐bodied active hunters were also most dominant, compared to upstream and downstream reaches. Overall, our study improves the understanding of how longitudinal environmental gradients and local habitat conditions jointly shape riparian communities under near‐natural conditions and may help conservation by providing reference information for the design of management actions.

## Author Contributions

**Léa Fargette:** conceptualization (equal), data curation (equal), formal analysis (equal), investigation (equal), methodology (equal), validation (equal), visualization (lead), writing – original draft (lead). **Martin H. Entling:** resources (equal), supervision (equal), validation (equal), writing – review and editing (equal). **Felicia Hoffmann:** investigation (equal), validation (equal), writing – review and editing (equal). **Alessandro Manfrin:** supervision (equal), validation (equal), writing – review and editing (equal). **Franziska Middendorf:** conceptualization (equal), investigation (equal), validation (equal), writing – original draft (equal). **Jens Schirmel:** conceptualization (equal), data curation (equal), methodology (equal), supervision (equal), validation (equal), writing – review and editing (lead).

## Funding

The study was funded by the Deutsche Forschungsgemeinschaft (DFG, German Research Foundation)—326210499/GRK2360.

## Conflicts of Interest

The authors declare no conflicts of interest.

## Supporting information


**Figure S1:** Number of pitfall traps with at least one adult individual per site across fifteen sampling sites in May (a) and September (b).

**Table S1:** Comparison of the number of individuals and species richness across catching methods and sampling times.

**Table S2:** Trait information across four trait categories for all collected species. Shading preference is represented by a numerical value from 0 to 1, ranging from open‐ to shaded‐habitat preference; moisture preference by a numerical value from 0 to 1, ranging from moist to dry habitats; body size by female body length in cm; and hunting guild as a categorical variable (active hunter or web builder), with species assigned based on multiple trait datasets.

**Table S3:** Spearman's rank correlations between environmental variables. Significant correlations are indicated in bold.

**Table S4:** Correlation values between environmental variables and the first two PCA axes for aquatic and terrestrial environmental datasets.

**Table S5:** Raw data used for the pitfall dataset including species information and environmental factors.

**Table S6:** Raw data used for the hand‐catch dataset including species information and environmental factors.

**Table S7:** Number of individuals and proportion (as percentages) of each collected species across stream sections for the pitfall, hand‐catch and total datasets.

## Data Availability

Data are available as supporting material in the form of appendix tables (Tables S5 and S6).
